# Safety of Pediatric rhGH Therapy: An Overview and the Need for Long-Term Surveillance

**DOI:** 10.3389/fendo.2021.811846

**Published:** 2021-12-24

**Authors:** Stefano Cianfarani

**Affiliations:** ^1^ Department of Systems Medicine, University of Rome Tor Vergata, Rome, Italy; ^2^ Dipartimento Pediatrico Universitario Ospedaliero, IRCCS “Bambino Gesù” Children’s Hospital, Rome, Italy; ^3^ Department of Women’s and Children’s Health, Karolinska Institute and University Hospital, Stockholm, Sweden

**Keywords:** growth hormome, GH deficiency (GHD), IGF - I, gh therapy, hypopituitarism

## Abstract

Growth hormone (GH) therapy dates back to 1958 and, though has shown an excellent safety profile in the short-term, has never ceased to raise concern about potential long-term side effects. In the last decade, a number of observational studies in different cohorts of young adult patients treated with GH during childhood have yielded conflicting results. The attention has mainly focused on three major potential risks associated with GH therapy: cancer, cardio and cerebrovascular diseases and diabetes. This review intends to provide a detailed overview of the main studies reporting long-term safety in subjects treated with rhGH therapy during childhood, highlighting the evidence for or against the risk of cancer, cardio and cerebrovascular diseases and diabetes.

## Introduction

Growth Hormone (GH) was initially purified from ox pituitaries (1) and thereafter successfully introduced in the treatment of children with GH deficiency in the middle of the last century (2) Human GH (hGH) was extracted from human pituitaries (pit-hGH) making it extremely difficult to find and stimulating the establishment of national agencies in many countries with the purpose to collect human pituitaries for extracting, purifying and distributing pit-hGH for the treatment of children with GH deficiency. Due to the shortage of raw material (i.e. human pituitaries), the therapeutic regimen of pit-hGH was far from optimal, being based on two to three intramuscular injections per week. Nevertheless, pit-hGH replacement therapy was extremely effective in inducing a robust and prolonged catch-up growth in children with hypopituitarism (3, 4).

pit-hGH therapy was continued until 1985 when the first three cases of Creutzfeldt—Jakob disease in young patients who had received pit-hGH injections during childhood were reported (5, 6). The cause of Creutzfeldt—Jakob disease is an infectious agent, termed prion, a misfolded protein able to induce neurodegeneration. As Creutzfeldt—Jakob disease usually affects older adults, these cases raised the suspicion of contamination of pit-hGH. This suspicion was later confirmed and between 1985 and 2003, over 200 cases were reported, mainly in France, United Kingdom, and United States (7).

Meanwhile, recombinant technology developed (8) and, in 1985, the first recombinant human GH (rhGH) produced from E. coli was approved by the FDA in the USA for the treatment of GH deficiency in childhood. Due to the safety concerns, pit-hGH was banned from the market and replaced by rhGH.

The virtually unlimited supply of rhGH led to the expansion of indications for rhGH therapy, now including childhood and adult GH deficiency, Turner syndrome, chronic renal failure, small for gestational age (SGA), Prader–Willi syndrome, Noonan syndrome, SHOX deficiency, idiopathic short stature (ISS), achondroplasia, short bowel syndrome and HIV wasting syndrome (9). This expansion of rhGH indications has not been associated with increased incidence of serious side effects. However, it has to be pointed out that the vast majority of available observational studies reporting on rhGH safety are short-term and not independent of Pharmaceutical Companies.

The aim of this review is to provide an overview of the main studies reporting long-term safety in subjects treated with rhGH therapy during childhood, focusing on the three major long-term concerns regarding rhGH therapy, namely cancer, cardio and cerebrovascular diseases and diabetes.

## Cancer Risk in Patients Treated With rhGH During Childhood

The experimental evidence supporting a role of GH and insulin-like growth factors (IGF-I and -II) in the development, expansion and dissemination of tumors is robust and based on countless data obtained in cell lines and animals, reported in detail in previous reviews (10–13). The epidemiological evidence linking circulating levels of IGF-I with increased risk of certain tumors, though less robust than experimental data, supports the role of exposure to high levels of IGFs in cancer risk (10). Consistently, the observation that congenital IGF-I deficiency confers protection from cancer, clearly indicates that low IGF-I levels are associated with reduced cancer risk (14–16).

Concern about the risk of cancer in children treated with GH was raised for the first time by case reports describing children who developed leukemia during or following GH treatment in Japan (17–19). Later analysis showed that at least half of these patients had conditions predisposing to leukemia, thus leading to overestimation of the frequency of malignancy (20). On the other hand, the national Cooperative Growth study, a nationwide study in USA initiated in 1985, did not show an increased risk of leukemia in children treated with GH (21).

Nevertheless, concern over a potential increase in cancer risk associated with GH therapy stimulated further observational studies. In 2002, a long-term study reporting data from 1,848 patients treated with pit-hGH during childhood and early adulthood, showed an increased risk of colorectal cancer and Hodgkin lymphoma (HL) (22). However, the absolute number of recorded deaths and cases was extremely low, though statistically significant (2 deaths for colorectal cancer with an expected number of 0.19 and two deaths for HL with an expected number of 0.18). Moreover, almost half of the study cohort had conditions different from idiopathic GH deficiency, including neoplasms and diseases predisposing to cancer.

In 2009, an EU funded (FP7-HEALTH) consortium of eight European countries (Safety and Appropriateness of GH treatments in Europe, SAGHE) was established with the purpose of evaluating long-term safety of rhGH therapy in childhood (23).

In 2012, preliminary and opposite results from different SAGHE cohorts were published (24, 25). In the French cohort comprising 6,500 young adult subjects treated with rhGH during childhood for the indications of isolated GH deficiency (IGHD), short stature associated with small for gestational age (SGA), or idiopathic short stature (ISS) a significant increase in mortality for bone was observed (24). On the contrary, in the same diagnostic cohorts (overall 2,500 patients) from Belgium, Sweden and The Netherlands, not a single case of death from cancer was observed (25).

In 2014, we carried out a systematic review and meta-analysis of studies reporting long-term safety data of rhGH therapy during childhood (26). The standard mortality ratio (SMR) for cancer was not significantly increased whereas overall cancer standard incidence ratio (SIR, 2.74; 95% confidence interval [CI], 1.18–4.41) was higher than reference populations. However, the analysis was based only on few available studies that, in addition, were affected by a number of confounders and biases.

In the GeNeSIS (Genetics and Neuroendocrinology of Short Stature International Study) observational study sponsored by Eli Lilly and conducted on more than 20,000 rhGH-treated patients with different diagnoses, no significant increase in cancer mortality was observed in IGHD, ISS, and SGA patients (27). It has to be pointed out that mean duration of follow-up in this study was 4.2 years only.

The study reporting mortality and morbidity for cancer from the entire dataset of all eight countries of the SAGHE consortium was published in 2017 (28). The patients were classified into three different classes of risk: (1) low risk: isolated growth failure, including IGHD, ISS and SGA; (2) high risk: including patients with previous history of cancer; (3) intermediate risk: non-isolated growth failure and non-cancer patients, including all the other patients with different diagnoses (Turner syndrome, Noonan syndrome etc.). 23,984 patients were enrolled for cancer mortality risk and 10,406 for cancer incidence. The average follow-up time for mortality was 16.5 years per patient, and for cancer incidence 14.8 years per patient. Both mortality and morbidity for cancer were not increased in the low risk cohort whereas SMR and SIR for almost all types of cancers were significantly increased in the high-risk group. The incidence of bone and bladder cancers was significantly raised in the intermediate risk cohort. No relationship between cancer risk and duration or cumulative dose of rhGH was found. In the high-risk cohort, cancer mortality risk increased significantly with increasing daily rhGH dose. Finally, the incidence of HL increased with time (28).

The French SAGHE cohort was then examined in a separate publication (29). Patients were followed for an average of 17.4 ± 5.3 years to a mean age of 28.4 ± 6.2 years.The overall incidence and mortality of cancer were not increased with the exception of bone tumors (SIR 3.5, 95% CI 1.1- 8.1; SMR 5.0, 95% CI 1.0- 14.6).

The study reporting data from the entire SAGHE cohort with more than 400 000 patient-years and up to 25 years of follow-up, focused on long-term overall and cause-specific mortality in young adult patients treated with recombinant human growth hormone during childhood (30). This study showed no increase in mortality for neoplasms in the low risk group (IGHD, ISS and SGA patients).

## Final Remarks on Cancer Risk in Patients Treated With rhGH During Childhood

The majority of observational studies, with the exception of the French SAGHE cohort, do not indicate that rhGH therapy affects the risk of cancer in children without other risk factors at least in the 15-16 years following rhGH therapy (**Table 1**) (31). All the available reports are affected by a series of confounders and biases. The study cohorts are often heterogenous, relatively small and observed over a short follow-up. The quality of study designs is different, some reporting data from death certificates and other from detailed analysis of clinical records. Moreover, the absolute rate of events is low, untreated control cohorts are not available, data regarding familial predisposition to cancer and exposure to environmental hazards are lacking as well as local cancer mortality and morbidity indices and information on rhGH dose and treatment duration. Therefore, it is still impossible to draw definitive conclusions on the basis of the available evidence.

**Table 1:** Summary of the available evidence of cancer, cardio-cerebrovascular and diabetes risk in young adulthood associated with rhGH therapy in childhood.

Cancer risk	Cardio-cerebrovascular risk	Type 2 Diabetes risk
I. **No evidence of increased risk in low risk group (IGHD, ISS and SGA).** II. **Increased risk of bone tumors in the French SAGHE cohort.**	**Evidence of potential increased risk, presumably in association with other risk factors such as family history, environment, lifestyle, ethnicity, comorbidities and, possibly, female gender.**	**Evidence of potential increased risk in presence of other risk factors such as obesity, family history, sedentary lifestyle, comorbidities.**

IGHD, isolated GH deficiency; ISS, idiopathic short stature; SGA, small for gestational age.

There is evidence that GH and IGF-I are not able to directly induce cell transformation and carcinogenesis but may amplify the DNA damaging effects induced by other factors (32, 33). On the other hand, experimental evidence suggests that both GH and IGF-I play a pivotal role in the expansion and dissemination of many tumors thus suggesting a possible accelerator effect in patients who have early stage neoplasms. This potentiality raises concern about the safety of rhGH treatment in patients with previous history of neoplasia, conditions predisposing to cancer (for instance RASopathies including Noonan syndrome) and chromosomal breakage syndromes or DNA-repair disorders, including Fanconi anemia, Bloom syndrome and Down syndrome.

In conclusion, long-term cancer surveillance is still needed in all patients treated with rhGH, especially in those with conditions predisposing to cancer risk and, more in general, in patients who received pharmacological rather than replacement rhGH therapy.

## Cardio and Cerebrovascular Risk in Patients Treated With rhGH During Childhood

Acromegaly, a disease characterized by excessive GH secretion, is associated with cardiovascular and cerebrovascular diseases (34). Though acromegalic patients have multiple risk factors that contribute to morbidity and mortality for cardiovascular diseases (CVD) such as hypertension, diabetes and dyslipidemia, nevertheless GH/IGF-1 excess per se may play a role in the increased CVD risk of these patients. Indeed, GH/IGF-1 excess may directly affect endothelial function *via* different mechanisms including: a) endothelial proliferation; b) dysfunction of endothelial progenitor cells; c) induction of oxidative stress; and d) reduction of oxidative defenses (35, 36). Two prospective Dutch and UK-based cohort studies in the elderly and adults respectively, have shown a U-shaped relationship between IGF-I levels and mortality, high circulating IGF-I levels being associated with increased risk of all-cause and CVD mortality (37, 38).

In a Dutch cohort of adult GHD patients on treatment with rhGH, CVD mortality was not increased (39). Data from KIMS, a global, multicenter, non-interventional, pharmaco-epidemiological study in which data were collected from GHD adults receiving rhGH replacement therapy, showed that mortality was slightly but significantly increased especially in women (40). Interestingly, standard mortality ratio (SMR) was significantly associated with IGF-I SDS and among the causes of death, mortality for cerebrovascular disease was significantly increased.

The first study showing increased cardio and cerebrovascular mortality in young adults treated with rhGH during childhood reported data of the French cohort of the SAGHE study (24). The cohort consisted of 6928 young adults with IGHD, neurosecretory dysfunction, ISS and born SGA. SMR was significantly increased for diseases of the circulatory system (SMR 3.07, 95% CI 1.40–5.83) or subarachnoid or intracerebral hemorrhage (SMR 6.66, 95% CI 1.79 –17.05). In contrast to these results, an observational study reporting mortality data from 2543 young adults recruited in the SAGHE cohorts from Belgium, The Netherlands and Sweden, with the same diagnostic categories of the French cohort, showed not a single case of death for cardio or cerebrovascular diseases (25).

A further report from the French SAGHE study group showed increased cerebrovascular morbidity for hemorrhagic stroke and particularly subarachnoid hemorrhage in 6874 young adults treated with rhGH during childhood for IGHD, ISS and SGA (41).

The study reporting mortality data from the complete dataset of all eight countries of the SAGHE consortium including more than 24000 patients with up to 25 years of follow-up was published in 2020 (30). The results showed that all-cause mortality was not increased in low-risk patients (IGHD and ISS) whereas it was significantly increased in children born small for gestational age, though this result was skewed by the French sub-cohort. Overall mortality was not associated with mean daily or cumulative doses of rhGH for any of the risk groups. Notably, when looking at cause specific mortality, mortality for diseases of circulatory system was significantly increased in all risk groups. A recent large nationwide cohort study conducted in Sweden, included patients treated with rhGH for the indications of IGHD, ISS and SGA (42). The Authors collected data on cardiovascular risk as well as a number of covariates such as gestational age, birth weight, birth length, socioeconomic status, and height. 53,444 individuals (3408 patients and 50036 controls) were followed up for a median of 14.9 years. The adjusted hazard ratio (HR) for all cardiovascular events was significantly increased in patients (HR, 1.69; 95% CI, 1.30-2.19), and particularly in women (HR, 2.05; 95% CI, 1.31-3.20). Each diagnostic category (i.e. IGHD, ISS and SGA) showed increased HRs. Interestingly, a higher risk of cardiovascular disease was associated with longer duration of rhGH treatment and total cumulative dose (42).

## Final Remarks on Cardio and Cerebrovascular Risk in Patients Treated With rhGH During Childhood

Although GH treatment has been reported to improve cardiovascular risk factors in GHD patients (43), the available data suggest a slight but significant increased cardio and cerebrovascular risk in patients treated with rhGH during childhood (**Table 1**). As mentioned in regard to cancer risk all the reports are burdened by many confounders and biases which prevent from drawing definitive conclusions. It is plausible that GH therapy in association with other risk factors such as genetics, environment, lifestyle, ethnicity, and comorbidities may concur in increasing cardio and cerebrovascular risk.

It has to be pointed out that the association between short stature and cardiovascular risk is well known (44). A genetic approach based on 180 height-associated genetic variants showed that a change in genetically determined height of 1 SD (6.5 cm) was associated with an increase of 13.5% (confidence interval, 5.4 to 22.1) in the risk of coronary artery disease (CAD) (45). In the same study, pathways linking height-associated genes with the risk of CAD were identified. These findings suggest that certain forms of short stature may per se be associated with increased cardio and cerebrovascular risk and the disentanglement of the potential adverse effect of hGH therapy from underlying predisposing factors still remains a major challenge.

## Diabetes Risk in Patients Treated With rhGH During Childhood

The role played by GH in glucose metabolism is well recognized. In particular, the administration of GH decreases glucose uptake and glucose oxidation, increases gluconeogenesis and reduces insulin sensitivity (46, 47).

The first report showing an association between rhGH therapy and risk of diabetes collected data of a large international pharmaco-epidemiological survey for monitoring efficacy and safety of GH therapy in children and adolescents (KIGS) (48). The incidence of type 1 diabetes was not increased in children treated with rhGH whereas the incidence of type 2 diabetes was six-fold higher than expected and diabetes persisted even after discontinuation of rhGH therapy. This finding was confirmed by another multinational observational study of children with growth disorders (GENESIS) which reported a significant higher incidence of type 2 diabetes in children treated with rhGH (49). Risk factors for type 2 diabetes were identified in 10 out of the 11 patients who developed the disease. A further report from GENESIS observational study on a larger population confirmed the increased risk of type 2 diabetes in patients treated with rhGH and with other predisposing factors (27).

In contrast to these findings a French study of the prevalence of diabetes in more than 5000 patients of patients treated with rhGH during childhood, showed no increased risk of diabetes in subjects treated with rhGH (50).

## Final Remarks on Diabetes Risk in Patients Treated With rhGH During Childhood

The majority of available data suggest that rhGH therapy is associated with increased risk of type 2 diabetes in patients with risk factors such as obesity, genetic predisposition and sedentary lifestyle (**Table 1**). Furthermore, rhGH therapy may function as an accelerator in the development of diabetes in patients with predisposing diseases such as Turner syndrome and organic GHD. Subjects born small for gestational age represent another group of patients potentially at risk of type 2 diabetes and metabolic syndrome, however, to date, the evidence on long-term metabolic safety of rhGH therapy in these subjects is reassuring (51, 52).

## Author Contributions

The author confirms being the sole contributor of this work and has approved it for publication.

## Conflict of Interest

The author declares that the research was conducted in the absence of any commercial or financial relationships that could be construed as a potential conflict of interest.

## Publisher’s Note

All claims expressed in this article are solely those of the authors and do not necessarily represent those of their affiliated organizations, or those of the publisher, the editors and the reviewers. Any product that may be evaluated in this article, or claim that may be made by its manufacturer, is not guaranteed or endorsed by the publisher.

## References

[1] Li CH Evans HM. The Isolation of Pituitary Growth Hormone. Science (1944) 99:183–4. doi: 10.1126/science.99.2566.183 17736901

[2] Raben MS. Treatment of a Pituitary Dwarf With Human Growth Hormone. J Clin Endocrinol Metab (1958) 18:901–3. doi: 10.1210/jcem-18-8-901 13563618

[3] Prader A Zachmann M Poley JR Illig R Szeky J. Long-Term Treatment With Human Growth Hormone (Raben) in Small Doses. Eval 18 Hypopituitary Patients Helv Paediatr Acta (1967) 22:423–40. 5595745

[4] Soyka LF Ziskind A Crawford JD. Treatment of Short Stature in Children and Adolescents With Human Pituitary Growth Hormone (Raben). N Engl J Med (1964) 271:754–64. doi: 10.1056/NEJM196410082711502 14186197

[5] Koch TK Berg BO De Armond SJ Gravina RF. Creutzfeldt-Jakob Disease in a Young Adult With Idiopathic Hypopituitarism. Possible Relation to the Administration of Cadaveric Human Growth Hormone. N Engl J Med (1985) 313:731–3. doi: 10.1056/NEJM198509193131206 3897861

[6] Centers for Disease C. Fatal Degenerative Neurologic Disease in Patients Who Received Pituitary-Derived Human Growth Hormone. MMWR Morb Mortal Wkly Rep (1985) 34:359–60, 365-6. 3923319

[7] Douet JY Huor A Cassard H Lugan S Aron N Mesic C. Prion Strains Associated With Iatrogenic CJD in French and UK Human Growth Hormone Recipients. Acta Neuropathol Com 9 (2021) 9:145. doi: 10.1186/s40478-021-01247-x PMC8403347 34454616

[8] Goeddel DV Heyneker HL Hozumi T Arentzen R Itakura K Yansura DG. Direct Expression in Escherichia Coli of a DNA Sequence Coding for Human Growth Hormone. Nature (1979) 281:544–8. doi: 10.1038/281544a0 386136

[9] Ranke MB Wit JM. Growth Hormone - Past, Present and Future. Nat Rev Endocrinol (2018) 14:285–300. doi: 10.1038/nrendo.2018.22 29546874

[10] Clayton PE Banerjee I Murray PG Renehan AG. Growth Hormone, the Insulin-Like Growth Factor Axis, Insulin and Cancer Risk. Nat Rev Endocrinol (2011) 7:11–24. doi: 10.1038/nrendo.2010.171 20956999

[11] Gallagher EJ LeRoith D. Minireview: IGF, Insulin, and Cancer. Endocrinology (2011) 152:2546–51. doi: 10.1210/en.2011-0231 21540285

[12] Chesnokova V Melmed S. Growth Hormone in the Tumor Microenvironment. Arch Endocrinol Metab (2019) 63:568–75. doi: 10.20945/2359-3997000000186 PMC7025769 31939481

[13] Boguszewski CL Boguszewski M. Growth Hormone's Links to Cancer. Endocr Rev (2019) 40:558–74. doi: 10.1210/er.2018-00166 30500870

[14] Steuerman R Shevah O Laron Z. Congenital IGF1 Deficiency Tends to Confer Protection Against Post-Natal Development of Malignancies. Eur J Endocrinol (2011) 164:485–9. doi: 10.1530/EJE-10-0859 21292919

[15] Guevara-Aguirre J Balasubramanian P Guevara-Aguirre M Wei M Madia F Cheng CW. Growth Hormone Receptor Deficiency Is Associated With a Major Reduction in Pro-Aging Signaling, Cancer, and Diabetes in Humans. Sci Transl Med (2011) 30:70ra13. doi: 10.1126/scitranslmed.3001845 PMC3357623 21325617

[16] Shevah O Laron Z. Patients With Congenital Deficiency of IGF-I Seem Protected From the Development of Malignancies: A Preliminary Report. Growth Horm IGF Res (2007) 17:54–7. doi: 10.1016/j.ghir.2006.10.007 17166755

[17] Endo M Kaneko Y Shikano T Minami H Chino J. Possible Association of Human Growth Hormone Treatment With an Occurrence of Acute Myeloblastic Leukemia With an Inversion of Chromosome 3 in a Child of Pituitary Dwarfism. Med Pediatr Oncol (1988) 16:45–7. doi: 10.1002/mpo.2950160111 3422334

[18] Hara T Komiyama A Ono H Akabane T. Acute Lymphoblastic Leukemia in a Patient With Pituitary Dwarfism Under Treatment With Growth Hormone. Acta Paediatr Jpn (1989) 31:73–7. doi: 10.1111/j.1442-200X.1989.tb01272.x 2504031

[19] Wada E Murata M Watanabe S. Acute Lymphoblastic Leukemia Following Treatment With Human Growth Hormone in a Boy With Possible Preanemic Fanconi's Anemia. Jpn J Clin Oncol (1989) 19:36–9. 2921816

[20] Stahnke N. Leukemia in Growth-Hormone-Treated Patients: An Update, 1992. Horm Res (1992) 38 Suppl 1:56–62. doi: 10.1159/000182571 1295814

[21] Bell J Parker KL Swinford RD Hoffman AR Maneatis T Lippe B. Long-Term Safety of Recombinant Human Growth Hormone in Children. J Clin Endocrinol Metab (2010) 95:167–77. doi: 10.1210/jc.2009-0178 19906787

[22] Swerdlow AJ Higgins CD Adlard P Preece MA. Risk of Cancer in Patients Treated With Human Pituitary Growth Hormone in the UK, 1959-85: A Cohort Study. Lancet (2002) 360:273–7. doi: 10.1016/S0140-6736(02)09519-3 12147369

[23] Swerdlow AJ Cooke R Albertsson-Wikland K Borgstrom B Butler G Cianfarani S. Description of the SAGhE Cohort: A Large European Study of Mortality and Cancer Incidence Risks After Childhood Treatment With Recombinant Growth Hormone. Horm Res Paediatr (2015) 84:172–83. doi: 10.1159/000435856 PMC4611066 26227295

[24] Carel JC Ecosse E Landier F Meguellati-Hakkas D Kaguelidou F Rey G. Long-Term Mortality After Recombinant Growth Hormone Treatment for Isolated Growth Hormone Deficiency or Childhood Short Stature: Preliminary Report of the French SAGhE Study. J Clin Endocrinol Metab (2012) 97:416–25. doi: 10.1210/jc.2011-1995 22238382

[25] Savendahl L Maes M Albertsson-Wikland K Borgstrom B Carel JC Henrard S. Long-Term Mortality and Causes of Death in Isolated GHD, ISS, and SGA Patients Treated With Recombinant Growth Hormone During Childhood in Belgium, The Netherlands, and Sweden: Preliminary Report of 3 Countries Participating in the EU SAGhE Study. J Clin Endocrinol Metab (2012) 97:E213–7. doi: 10.1210/jc.2011-2882 22238393

[26] Deodati A Ferroli BB Cianfarani S. Association Between Growth Hormone Therapy and Mortality, Cancer and Cardiovascular Risk: Systematic Review and Meta-Analysis. Growth Hormone Igf Res (2014) 24:105–11. doi: 10.1016/j.ghir.2014.02.001 24818783

[27] Child CJ Zimmermann AG Chrousos GP Cummings E Deal CL Hasegawa T. Safety Outcomes During Pediatric GH Therapy: Final Results From the Prospective GeNeSIS Observational Program. J Clin Endocrinol Metab (2019) 104:379–89. doi: 10.1210/jc.2018-01189 PMC6300411 30219920

[28] Swerdlow AJ Cooke R Beckers D Borgstrom B Butler G Carel JC. Cancer Risks in Patients Treated With Growth Hormone in Childhood: The SAGhE European Cohort Study. J Clin Endocr Metab (2017) 102:1661–72. doi: 10.1210/jc.2016-2046 PMC6061931 28187225

[29] Poidvin A Carel JC Ecosse E Levy D Michon J Coste J. Increased Risk of Bone Tumors After Growth Hormone Treatment in Childhood: A Population-Based Cohort Study in France. Cancer Med (2018) 7:3465–73. doi: 10.1002/cam4.1602 PMC6051149 29905027

[30] Savendahl L Cooke R Tidblad A Beckers D Butler G Cianfarani S. Long-Term Mortality After Childhood Growth Hormone Treatment: The SAGhE Cohort Study. Lancet Diabetes Endocrinol (2020) 8:683–92. doi: 10.1016/S2213-8587(20)30163-7 32707116

[31] Allen DB Backeljauw P Bidlingmaier M Biller BMK Boguszewski M Burman P. GH Safety Workshop Position Paper: A Critical Appraisal of Recombinant Human GH Therapy in Children and Adults. Eur J Endocrinol (2016) 174:P1–9. doi: 10.1530/EJE-15-0873 PMC4674592 26563978

[32] Tedeschi B Spadoni GL Sanna ML Vernole P Caporossi D Cianfarani S. Increased Chromosome Fragility in Lymphocytes of Short Normal Children Treated With Recombinant Human Growth Hormone. Hum Genet (1993) 91:459–63. doi: 10.1007/BF00217772 7686129

[33] Cianfarani S Tedeschi B Germani D Prete SP Rossi P Vernole P. *In Vitro* Effects of Growth Hormone (GH) and Insulin-Like Growth Factor I and II (IGF-I and -II) on Chromosome Fragility and P53 Protein Expression in Human Lymphocytes. Eur J Clin Invest (1998) 28:41–7. doi: 10.1046/j.1365-2362.1998.00247.x 9502186

[34] Kasuki L Antunes X Lamback EB Gadelha MR. Acromegaly: Update on Management and Long-Term Morbidities. Endocrinol Metab Clin North Am (2020) 49:475–86. doi: 10.1016/j.ecl.2020.05.007 32741483

[35] Wolters TLC Netea MG Riksen NP Hermus A Netea-Maier RT. Acromegaly, Inflammation and Cardiovascular Disease: A Review. Rev Endocr Metab Disord (2020) 21:547–68. doi: 10.1007/s11154-020-09560-x PMC7560935 32458292

[36] Maffei P Dassie F Wennberg A Parolin M Vettor R. The Endothelium in Acromegaly. Front Endocrinol (Lausanne) (2019) 10:437. doi: 10.3389/fendo.2019.00437 31396153 PMC6667653

[37] van Bunderen CC van Nieuwpoort IC van Schoor NM Deeg DJ Lips P Drent ML. The Association of Serum Insulin-Like Growth Factor-I With Mortality, Cardiovascular Disease, and Cancer in the Elderly: A Population-Based Study. J Clin Endocrinol Metab (2010) 95:4616–24. doi: 10.1210/jc.2010-0940 20610588

[38] Xie Y Huang C Zhu X Wang J Fan X Fu Z. Association Between Circulating Insulin-Like Growth Factor 1 and Risk of All-Cause and Cause-Specific Mortality. Eur J Endocrinol (2021) 185:681–9. doi: 10.1530/EJE-21-0573 34478404

[39] van Bunderen CC van Nieuwpoort IC Arwert LI Heymans MW Franken AA Koppeschaar HP. Does Growth Hormone Replacement Therapy Reduce Mortality in Adults With Growth Hormone Deficiency? Data From the Dutch National Registry of Growth Hormone Treatment in Adults. J Clin Endocrinol Metab (2011) 96:3151–9. doi: 10.1210/jc.2011-1215 21849531

[40] Gaillard RC Mattsson AF Akerblad AC Bengtsson B Cara J Feldt-Rasmussen U. Overall and Cause-Specific Mortality in GH-Deficient Adults on GH Replacement. Eur J Endocrinol (2012) 166:1069–77. doi: 10.1530/EJE-11-1028 22457236

[41] Poidvin A Touze E Ecosse E Landier F Bejot Y Giroud M. Growth Hormone Treatment for Childhood Short Stature and Risk of Stroke in Early Adulthood. Neurology (2014) 83:780–6. doi: 10.1212/WNL.0000000000000737 25122206

[42] Tidblad A Bottai M Kieler H Albertsson-Wikland K Savendahl L. Association of Childhood Growth Hormone Treatment With Long-Term Cardiovascular Morbidity. JAMA Pediatr (2021) 175:e205199. doi: 10.1001/jamapediatrics.2020.5199 33346824 PMC7754074

[43] Gazzaruso C Gola M Karamouzis I Giubbini R Giustina A. Cardiovascular Risk in Adult Patients With Growth Hormone (GH) Deficiency and Following Substitution With GH–An Update. J Clin Endocrinol Metab (2014) 99:18–29. doi: 10.1210/jc.2013-2394 24217903

[44] Rich-Edwards JW Manson JE Stampfer MJ Colditz GA Willett WC Rosner B. Height and the Risk of Cardiovascular Disease in Women. Am J Epidemiol (1995) 142:909–17. doi: 10.1093/oxfordjournals.aje.a117738 7572971

[45] Nelson CP Hamby SE Saleheen D Hopewell JC Zeng L Assimes TL. Genetically Determined Height and Coronary Artery Disease. N Engl J Med (2015) 372:1608–18. doi: 10.1056/NEJMoa1404881 PMC4648271 25853659

[46] Weaver JU Monson JP Noonan K John WG Edwards A Evans KA. The Effect of Low Dose Recombinant Human Growth Hormone Replacement on Regional Fat Distribution, Insulin Sensitivity, and Cardiovascular Risk Factors in Hypopituitary Adults. J Clin Endocrinol Metab (1995) 80:153–9. doi: 10.1210/jcem.80.1.7829604 7829604

[47] Moller N Jorgensen JO. Effects of Growth Hormone on Glucose, Lipid, and Protein Metabolism in Human Subjects. Endocr Rev (2009) 30:152–77. doi: 10.1210/er.2008-0027 19240267

[48] Cutfield WS Wilton P Bennmarker H Albertsson-Wikland K Chatelain P Ranke MB. Incidence of Diabetes Mellitus and Impaired Glucose Tolerance in Children and Adolescents Receiving Growth-Hormone Treatment. Lancet (2000) 355:610–3. doi: 10.1016/S0140-6736(99)04055-6 10696981

[49] Child CJ Zimmermann AG Scott RS Cutler GB Jr. Battelino T Blum WF. Prevalence and Incidence of Diabetes Mellitus in GH-Treated Children and Adolescents: Analysis From the GeNeSIS Observational Research Program. J Clin Endocrinol Metab (2011) 96:E1025–34. doi: 10.1210/jc.2010-3023 21490076

[50] Poidvin A Weill A Ecosse E Coste J Carel JC. Risk of Diabetes Treated in Early Adulthood After Growth Hormone Treatment of Short Stature in Childhood. J Clin Endocrinol Metab (2017) 102:1291–8. doi: 10.1210/jc.2016-3145 28324032

[51] Van Der Steen M Lem AJ van der Kaay DCM Hokken-Koelega ACS. Insulin Sensitivity and Beta-Cell Function in SGA Children Treated With GH and GnRHa: Results of a Long-Term Trial. J Clin Endocrinol Metab (2016) 101:705–13. doi: 10.1210/jc.2015-3435 26653111

[52] van der Steen M Smeets CCJ Kerkhof GF Hokken-Koelega ACS. Metabolic Health of Young Adults Who Were Born Small for Gestational Age and Treated With Growth Hormone, After Cessation of Growth Hormone Treatment: A 5-Year Longitudinal Study. Lancet Diabetes Endocrinol (2017) 5:106–16. doi: 10.1016/S2213-8587(16)30422-3 28011067

